# Mental Health of the Participants of the Third Age University Program: A Cross-Sectional Study

**DOI:** 10.3389/fpsyt.2020.00656

**Published:** 2020-07-10

**Authors:** Mateusz Cybulski, Łukasz Cybulski, Urszula Cwalina, Krystyna Kowalczuk, Elżbieta Krajewska-Kułak

**Affiliations:** ^1^ Department of Integrated Medical Care, Faculty of Health Sciences, Medical University of Bialystok, Bialystok, Poland; ^2^ Faculty of Social Sciences, University of Warmia and Mazury, Olsztyn, Poland; ^3^ Department of Statistics and Medical Informatics, Faculty of Health Sciences, Medical University of Bialystok, Bialystok, Poland

**Keywords:** alcohol addiction, anxiety, bipolar disorder, depression, emotion, insomnia, mood disorders, older adults

## Abstract

**Introduction:**

Population aging is a progressive demographic phenomenon observed in all countries worldwide. The progressive global process of population aging poses many threats, especially in the context of the mental health of the elderly. Third Age Universities are an essential preventive measure shown to improve the quality of life and psychological wellbeing of the elderly.

**Objectives:**

The aim of this study was to analyze the mental status of older persons attending Third Age Universities in Poland, with particular emphasis on sociodemographic sources of variance in psychological indices.

**Methods:**

The study included a group of the Third Age University program participants from Poland. A total of 247 persons were enrolled, among them 215 women and 32 men. The study was carried out as a diagnostic survey, using the following validated psychometric scales: The Mood Disorder Questionnaire (MDQ), Geriatric Depression Scale (GDS), General Health Questionnaire-28 (GHQ-28), The Athens Insomnia Scale (AIS), Courtauld Emotional Control Scale (CECS), State-Trait Anxiety Inventory (STAI) and SMAST-G—Short Michigan Alcoholism Screening Test—Geriatric Version.

**Results:**

The vast majority of the respondents did not screen positively for possible bipolar disorder. However, more than 90% of the participants presented with the symptoms of mild depression, and more than one-third had manifestations of non-psychotic mental morbidity. Nearly half of the respondents had complaints associated with insomnia, and in more than one-fourth, SMAST-G score raised suspicion of problem alcohol drinking. Retired participants were shown to present with significantly higher levels of anger control than the occupationally active respondents. Respondents with poor economic status had significantly higher levels of symptoms for non-psychotic mental disorders. Men significantly more often than women showed symptoms raising suspicion of alcohol-related problems.

**Discussion:**

In conclusion, the study group was characterized primarily by the mild depressive and anxiety symptoms. The mental health of the Polish participants of the Third Age University program was significantly modulated by their socio-occupational and marital status, and financial condition. The study showed that persons with likely problem alcohol drinking differed from other respondents in terms of the occurrence of possible bipolar disorder, depression, and non-psychotic symptoms of mental disorders, state and trait anxiety levels and anger control. There is a need for further research in the field of mental health status in the Third Age Universities seniors to determine the prevalence of these phenomena on a national scale.

## Introduction

Population aging, manifesting as an inevitable increase of the proportion of the elderly in population structure, is a progressive demographic phenomenon observed in all countries worldwide. According to the United Nations data, the global number of the elderly, i.e. persons aged 65 years or older reached 703 million in 2019. It is projected that until 2050, this number may increase more than twice, up to 1.5 billion (1). Further, a significant increase in the proportion of people older than 80 years is expected, from 137 million in 2017 to 425 million in 2050 (2). The demographic prognoses for Poland are equally alarming. In 2035, the proportion of Poles aged 65 years or older is projected at 25%, and by 2060, Poland is expected to be one of the oldest European communities (3). The progressive global process of population aging, particularly evident in Europe, poses many threats, especially in the context of the mental health of the elderly (4).

Among mental disorders of older age, depression seems to be the most widespread health problem which contributes to a substantial deterioration of the quality of life and an increase in healthcare expenditures (5, 6). The prevalence of depression among the elderly is estimated at 7% globally, whereas anxiety-related disorders and problems associated with alcohol abuse are reported in 3.8% and nearly 1% of the older population, respectively (7). The prevalence of sleeplessness was shown to be higher among the elderly than in younger persons, 30–48% vs. 12–20%, respectively (8).

Third Age Universities, popular across the world, also in Poland, are an essential preventive measure shown to improve the quality of life and psychological wellbeing of the elderly. In line with current gerontological theories, education may promote self-sufficiency and independence of the elderly through the improvement of their self-esteem, mental and physical health. Indeed, older persons who attend the Third Age Universities expect that participation in this initiative will contribute to a better perception of their social status, physical and mental health (9). Students of the Third Age Universities are an interesting group of seniors in terms of research. The progressing phenomenon of the aging of the society on a global scale, as well as the increasing socio-educational activity of seniors around the world means that students of this type of University are becoming the main group of older adults.

Considering all the above, the primary aim of this study was to analyze the mental status of older persons attending Third Age Universities in Poland, with particular emphasis on sociodemographic sources of variance in psychological indices. Moreover, we analyzed the prevalence of selected mental illnesses within the geriatric population of the Third Age University participants, using widely available validated psychometric scales; the use of the validated scales enabled us to compare our findings with the results of similar, representative studies carried out in other countries. Additionally, we analyzed correlations between the results obtained with various scales.

It needs to be stressed that equally important value of the present study was its exploratory potential. Our findings were compared with the results of a few previous Poland-wide studies, such as PolSenior examining the prevalence of depressive disorders in persons older than 65 years, and NATPOL analyzing the prevalence of the disorders of sleep.

We hypothesized that the prevalence of mental disorders within the population of the Third Age University participants is relatively high and tends to increase with age. Moreover, we expected depressive disorders to be the most common mental illness found in this specific geriatric population. To verify the hypotheses mentioned above, the following research questions were formulated:

1. What is the true prevalence of mental disorders among the elderly participating in the Third Age University program in Poland?
2. How the prevalence of mental disorders among the Third Age University participants varies depending on gender, place of residence, education, marital status and socio-occupational status?

## Materials and Methods

### Participants

The study included a group of the Third Age University program participants from Poland. A total of 247 persons were enrolled, among them 215 women and 32 men. Over half of the respondents (55.2%) were in the age range 60–69 years. A similar percentage of respondents (52.6%) were widowed. Over two-thirds of students of the Third Age Universities (67.2%) had higher education. A fairly even distribution of responses was recorded for the place of residence. The vast majority of respondents (94.3%) were retired. Detailed sociodemographic characteristics of the respondents are shown in **Table 1**.

**Table 1:** Sociodemographic characteristics of the respondents.

Sociodemographic value		N	%
**Gender**	men	32	13.0%
	women	215	87.0%
**Age**	60–69 years	138	55.9%
	70–79 years	105	42.5%
	80–89 years	4	1.6%
**Marital status**	married	130	52.6%
	widowed	52	21.%
	in separation	3	1.2%
	divorced	39	15.8%
	single	23	9.3%
**Education**	higher (completed bachelor studies)	166	67.2%
	secondary	66	26.7%
	technical	13	5.3%
	vocational	2	0.8%
**Place of residence**	village	29	11.8%
	town up to 50,000	68	27.5%
	town up to 200,000	68	27.5%
	city up to 500,000	15	6.1%
	city above 500,000	67	27.1%
**Economic status**	poor	6	2.4%
	moderate	100	40.5%
	good	120	48.6%
	very good	21	8.5%
**Socio-occupational status**	retired	233	94.3%
	disability pensioner	3	1.2%
	occupationally active	11	4.5%
**Voivodeship**	Lower Silesian	37	14.98%
	Kuyavian–Pomeranian	9	3.64%
	Lublin	32	12.96%
	Lubusz	1	0.40%
	Łódź	1	0.40%
	Lesser Poland	8	3.24%
	Mazovian	62	25.10%
	Opole	0	0.00%
	Subcarpathian	8	3.24%
	Podlasie	5	2.02%
	Pomeranian	22	8.91%
	Silesian	13	5.26%
	Holy Cross	0	0.00%
	Warmian–Masurian	5	2.02%
	Greater Poland	17	6.88%
	West Pomeranian	27	10.93%

### Study Design and Data Collection

A cross-sectional study was conducted between 1 June and 30 November 2019. The invitations were sent to official e-mail addresses published on the websites of all Third Age University chapters existing in Poland. In connection with the above, the snowball sampling was used in the study—a non-random sample selection method consisting in recruiting participants by other participants (most often by the Presidents of Third Age Universities or substantive coordinators on behalf of the Universities, at which these forms of education of the elderly are conducted).

The study was carried out with an online survey created with dedicated software (Webankieta). A link to the survey was included in the invitation e-mail sent to the address available on the official University websites. Respondents’ responses were recorded on the platform used and then downloaded as raw data prepared for statistical analysis. Mean time to complete the survey was 43 min.

Aside from age and enrollment in the Third Age University program, an additional inclusion criterion was written informed consent to participate in the study. The exclusion criteria were: (1) age ≤60 years, (2) lack of literacy skills (a respondent who had completed at least primary school could participate in the study) and (3) lack of written consent to participate in the study. Each participant could withdraw from the study at any time.

The respondents were chosen by nonprobability sampling. Referring to the total number of questionnaires returned, the rate of complete filling was 47.3%. The remaining percentage (52.7%) was incomplete questionnaires.

### Measures

#### The Mood Disorder Questionnaire (MDQ)

The Mood Disorder Questionnaire (MDQ) was designed as a screening instrument for possible bipolar disorder. Other than being used for research purposes, MDQ is also invaluable in everyday clinical practice as it can promptly and simply identify patients with possible bipolar disorder (10–12). MDQ is a self-report instrument completed by the patient or appropriately trained questioner. Time to complete the survey is estimated at 5–10 min. The instrument consists of three parts: a short symptom inventory (checklist), with 13 dichotomous (yes/no) questions about a history of mania or hypomania episodes. The inventory was developed based on the DSM-IV criteria for mania and hypomania. The second part includes only one question, whether the symptoms chosen from the inventory appeared synchronously or not. The aim of the third part of the scale is to verify to which degree social functioning of the respondent has been jeopardized by the symptoms chosen from the inventory. The overall MDQ score is obtained by summing up all “yes” responses from the symptom inventory (maximum score is 13 pts) (10–12). An adult respondent screens positive for possible bipolar disorder if at least seven “yes” responses to the questions about (hypo)mania episodes were chosen, and at least two of these episodes coincided. The third criterion that needs to be satisfied is “moderate problem” or “serious problem” response to the question about the impact of the symptoms on respondent’s functioning, included in the third part of the instrument. The sensitivity of the scale is 73.4%, whereas its reliability equals 89.9% (10, 13).

#### Geriatric Depression Scale (GDS)

Geriatric Depression Scale (GDS) was developed by Yesavage et al. in 1983 (14). The full version of the scale consists of 30 statements. GDS measures the severity of depression in the elderly (persons aged more than 60 years) during a week preceding the survey. It is a self-report scale with the choice between “yes” and “no” responses to short, comprehendible questions. The questions intentionally do not address somatic complaints or ailments. Each response is scored 0 or 1 pt, and hence, the overall score for the full version can range between 0 and 30 pts. The scores from 0 to 9 pts correspond to the lack of depression, whereas those from 10 to 19 pts and from 20 to 30 pts to mild and severe depression, respectively. The scale has good psychometric properties, with the sensitivity and specificity of 84 and 95%, respectively. Mean time to complete the survey is 20 min (14).

#### General Health Questionnaire-28 (GHQ-28)

GHQ is a screening instrument to assess psychological wellbeing in the general population (15). It is used to estimate the severity of non-psychotic psychiatric disorders and to identify persons at increased risk of psychiatric morbidity (16, 17). There are several versions of the instrument, the basic, long one with 60 items (GHQ-60), and shorter ones obtained by combining some questions. The GHQ-28 was developed as a result of factor analysis of the GHQ-60; aside from the general psychological wellbeing, this version considers also somatic symptoms, anxiety, depression, insomnia and social impairment (18). The self-report scale includes questions about life situation and the psychological condition of the respondent, each rated on a 4-point Likert-type scale. The responses are scored 0, 1, 2 or 3 pts, from left to right (19). The instrument provides information about general psychological wellbeing of the respondent, as well as about occurrence of some specific symptoms measured with four subscales: somatic symptoms—questions 1–7 (GHQ-28-A), anxiety and insomnia—questions 8–14 (GHQ-28-B), social impairment—questions 15–21 (GHQ-28-C), and depression—questions 22–28 (GHQ-28-D) (20). Maximum overall GHQ-28 score is 84 pts, and the cut-off value for possible non-psychotic psychiatric morbidity is 23/24 pts (21). Cronbach’s alpha for the scale was determined at 0.9–0.95 (22).

#### The Athens Insomnia Scale (AIS)

The Athens Insomnia Scale (AIS) is a short self-report scale with eight statements about various manifestations of insomnia, quantified based on ICD-10 criteria (23, 24). Each statement is rated on a scale from 0 to 3 pts, where 0 corresponds to the lack of a given symptom and 3 to its maximum severity. Hence, the overall AIS score can range between 0 and 24 pts. The first five items refer to the disturbances of sleep and sleep quality (sleep induction, awakenings during the night, final awakening, total sleep duration and sleep quality) and correspond to diagnostic criterion A for non-organic insomnia according to ICD-10. The respondent should choose a symptom if it appeared at least three times a week over a period of one month, which is consistent with diagnostic criterion B for insomnia according to ICD-10. The other three items refer to functioning during the day (wellbeing, functioning capacity, sleepiness) and correspond to diagnostic criterion C for insomnia according to ICD-10, i.e. the negative consequences of insomnia (25, 26). A validation study demonstrated high reliability and accuracy of the original version of the AIS. The overall score of ≥6 pts was shown to be a cut-off value identifying persons who are highly likely to suffer from insomnia (93% sensitivity, 85% specificity) (27, 28). AIS is one of the most commonly used scales, whether for diagnostic purposes or in research on insomnia treatments (27, 28).

#### Courtauld Emotional Control Scale (CECS)

Courtauld Emotional Control Scale (CECS) consists of three subscales, each with seven statements about the way of showing anger, depression and anxiety. The scale measures subjective control of anger, anxiety and depression in difficult situations and is dedicated to the examination of adults, whether healthy or diseased. The CECS is a self-report scale. Its global score, being a sum of the scores for all three subscales, is referred to as the overall index of emotion control (29, 30). The aim of examination with the CECS is to determine to which degree an individual is convinced subjectively of the ability to control his/her response after experiencing specific negative emotions. The overall index of emotion control can range between 21 and 84 pts. The higher the score, the greater the respondent’s ability to suppress negative emotions (29). The reliability of the Polish version of the scale was verified based on its internal consistency rate and absolute stability; Cronbach’s alpha values for the control of anger, depression and anxiety were 0.80, 0.77 and 0.78, respectively, whereas the alpha value for the overall index of emotion control was 0.87 (30).

#### State-Trait Anxiety Inventory (STAI)

Anxiety was assessed with the Polish version of the original Spielberger STAI, which is usually referred to as the STAI-X (31–33). The STAI-X is an extensively used self-administered inventory of two sections containing 20 items each, designed to explore anxiety in its temporary condition of “state anxiety” (STAI-X1) and the more general and long-standing quality of “trait anxiety”(STAI-X2) (31, 33). The STAI-X1 assesses how respondents feel “right now, at this moment”, and the STAI-X2 target show respondents “generally feel”. Each item is scored on a 4-point Likert scale, with choices ranging from 1 (“not at all”) to 4 (“very much so”) for the state scale, and 1 (“almost never”) to 4 (“almost always”) for the trait scale. The minimum score for each section is 20, with a maximum score of 80. A total score of 40 or more indicates an anxious condition. The higher the score is, the more severe the anxiety condition (31, 33). It is used as an indicator of general anxiety, general psychological distress, and general emotional distress (33). The reliability of the scale, measured as its internal consistency rate in a group of adult women and men varies between 0.76 and 0.92, and its theoretical accuracy for men and women is 0.51 and 0.57, respectively (31, 32).

#### SMAST-G—Short Michigan Alcoholism Screening Test—Geriatric Version

Short Michigan Alcoholism Screening Test—Geriatric Version **(**SMAST-G) is a validated test considered currently the best instrument for early detection of problem alcohol drinking in the elderly. The scale has high sensitivity and specificity (34). It consists of 10 close-ended questions:

When talking with others, do you ever underestimate how much you drink?
After a few drinks, have you sometimes not eaten or been able to skip a meal because you didn’t feel hungry?
Does having a few drinks help decrease your shakiness or tremors?
Does alcohol sometimes make it hard for you to remember parts of the day or night?
Do you usually take a drink to calm your nerves?
Do you drink to take your mind off your problems?
Have you ever increased your drinking after experiencing a loss in your life?
Has a doctor or nurse ever said they were worried or concerned about your drinking?
Have you ever made rules to manage your drinking?
When you feel lonely, does having a drink help? (34).

Answer “yes” to two or more questions implies that the respondent may experience an alcohol-related problem and should undergo further evaluation (34).

### Procedure and Ethical Considerations

The study was carried out in accordance with the recommendations, and was reviewed and approved by the Bioethics Committee of the Medical University in Bialystok (statute no. R-I-002/592/2019). All subjects gave the written informed consent in accordance with the Declaration of Helsinki.

### Statistical Analysis

The data were processed with Microsoft Excel 2013 spreadsheet and analyzed with Statistica Data Miner C QC PL package. The significance of relationships between qualitative variables was verified with Pearson’s chi-squared test (χ²). Normal distribution of quantitative variables was checked with Shapiro–Wilk W-test. As none of the variables was distributed normally, they were analyzed with non-parametric tests; the significance of differences between two groups was verified with Mann–Whitney U-test, and multiple groups were compared using Kruskal–Wallis ANOVA and appropriate post-hoc tests. Associations between pairs of quantitative variables were analyzed based on Spearman’s coefficients of rank correlation. The results of all tests were considered significant at p <0.05.

## Results

Mean MDQ score was 3.52 ± 2.87 pts, suggesting that the surveyed participants of the Third Age University program were unlikely to present with possible bipolar disorder. Mean GDS score was 12.75 ± 3.03 pts, which corresponded to a mild depression in the study respondents. Mean overall GHQ-28 score was 21.44 ± 10.21 pts, which implied that the participants of the study did not suffer from non-psychotic psychiatric disorders. Mean AIS score was 5.96 ± 4.12 pts, which means that the respondents were unlikely to suffer from insomnia. The mean level of state anxiety (STAI X-1) was 39.99 ± 10.00 pts, whereas the mean level of trait anxiety (STAI X-2) amounted to 39.19 ± 9.11 pts; these results suggest that the participants of the study presented with moderate levels of anxiety. Mean overall CECS score was 52.43 ± 8.41 pts, which is considered a moderate ability to suppress negative emotions. Regarding individual CECS subscales, mean levels of anger and depression control were slightly below 18 out of 28 possible pts, whereas the mean level of anxiety control was slightly less than 17 out of 28 pts. Mean SMAST-G score was 1.16 ± 1.79 pts, which was interpreted as the lack of alcohol-related problems in the study group. Detailed descriptive statistics for all the scales mentioned above are presented in **Table 2**.

**Table 2:** Descriptive statistics for the scales used in the study.

	$\bar{x}$	SD	Min.	Q_1_	Me	Q_3_	Max.
**MDQ**	3.52	2.87	0	1	3	5	13
**GDS**	12.75	3.03	5	11	12	14	30
**GHQ-28**	21.44	10.21	6	14	19	26	70
**AIS**	5.96	4.12	0	3	5	8	22
**STAI (X-1)**	39.33	10.00	20	32	40	45	77
**STAI (X-2)**	39.19	9.11	22	32	38	45	70
**CECS_anger**	17.94	3.69	7	15	18	20	28
**CECS_depression**	17.93	3.84	7	15	18	20	28
**CECS_anxiety**	16.57	2.83	7	15	17	19	24
**CECS_total**	52.43	8.41	21	46	52	58	74
**SMAST-G**	1.16	1.79	0	0	0	2	10

AIS, Athens Insomnia Scale; CECS, Courtauld Emotional Control Scale; GDS, Geriatric Depression Scale; GHQ-28, General Health Questionnaire-28; Max., maximum; MDQ, Mood Disorder Questionnaire; Me, median; Min., minimum; SD, standard deviation; SMAST-G, Short Michigan Alcoholism Screening Test–Geriatric Version; STAI, State-Trait Anxiety Inventory; Q_1_, lower quartile; Q_3_, upper quartile; $\bar{x}$, mean.

The vast majority of the respondents did not screen positively for possible bipolar disorder. However, more than 90% of the participants presented with the symptoms of mild depression, and more than one-third had manifestations of non-psychotic mental morbidity. Nearly half of the respondents had complaints associated with insomnia, and in more than one-fourth, SMAST-G score raised suspicion of problem alcohol drinking. Detailed results are shown in **Table 3**.

**Table 3:** Prevalence of various symptoms of mental disorders determined with the scales used in the study.

Mental disorder		N	%
**Possible bipolar disorder (MDQ)**	yes	10	4.05%
	no	237	95.95%
**Depressive symptoms (GDS)**	no	16	6.48%
	mild	223	90.28%
	moderate or severe	8	3.24%
**Symptoms of non-psychotic mental disorders** **(GHQ-28)**	yes	87	35.22%
	no	160	64.78%
**Insomnia symptoms** **(AIS)**	yes	109	44.13%
	no	138	55.87%
**Problem alcohol drinking** **(SMAST-G)**	yes	67	27.13%
	no	180	72.87%

AIS, Athens Insomnia Scale; GDS, Geriatric Depression Scale; GHQ-28, General Health Questionnaire-28; MDQ, Mood Disorder Questionnaire; SMAST-G, Short Michigan Alcoholism Screening Test—Geriatric Version.

Comparative analysis of the results stratified according to the socio-occupational status of the respondents did not include the recipients of disability pension as this group included only three persons. Retired participants were shown to present with significantly higher levels of anger control than the occupationally active respondents. The results obtained with other scales did not differ significantly depending on the socio-occupational status of the study subjects (**Table 4**).

**Table 4:** Effects of socio-occupational status on the scores of psychometric scales used in the study.

	Retired N = 233		Occupationally active N = 11		p
	$\bar{x}$	Me	$\bar{x}$	Me	
**MDQ**	3.55 ± 2.9	3.0	3.27 ± 2.49	3.0	NS
**GDS**	12.73 ± 3.06	12.0	13 ± 2.9	13.0	NS
**GHQ-28**	21.57 ± 10.32	19.0	18.73 ± 8.67	15.0	NS
**AIS**	6.09 ± 4.18	5.0	4.09 ± 2.07	5.0	NS
**STAI (X-1)**	39.54 ± 9.95	40.0	33.73 ± 10.58	33.0	NS
**STAI (X-2)**	39.43 ± 9.1	39.0	34.91 ± 9.24	34.0	NS
**CECS_anger**	18.06 ± 3.69	18.0	15.55 ± 3.17	15.0	0.019*
**CECS_depression**	17.91 ± 3.79	18.0	18.18 ± 5.06	16.0	NS
**CECS_anxiety**	16.59 ± 2.8	17.0	16.27 ± 2.97	16.0	NS
**CECS_total**	52.56 ± 8.31	52.0	50 ± 9.92	46.0	NS
**SMAST-G**	1.18 ± 1.82	0.0	0.82 ± 1.08	0.0	NS

AIS, Athens Insomnia Scale; CECS, Courtauld Emotional Control Scale; GDS, Geriatric Depression Scale; GHQ-28, General Health Questionnaire-28; MDQ, Mood Disorder Questionnaire; Me, median; NS, not significant; p, p-value; SD, standard deviation; SMAST-G, Short Michigan Alcoholism Screening Test—Geriatric Version; STAI, State-Trait Anxiety Inventory; $\bar{x}$, mean; *, statistically significant value (p < 0.05; Mann-Whitney U-test).

Based on the SMAST-G scores, 67 respondents were identified as prone to alcohol-related problems. Detailed analysis showed that these persons also presented with significantly higher MDQ scores, higher levels of depression, symptoms of non-psychotic mental disorders, state and trait anxiety, as well as with significantly lower levels of anger control than the other respondents (**Table 5**).

**Table 5:** Effect of possible problem alcohol drinking on the scores of psychometric scales used in the study.

	SMAST-G (2 pts and more) N = 67		SMAST-G (<2 pts) N = 180		p
	$\bar{x}$	Me	$\bar{x}$	Me	
**MDQ**	4.87 ± 3.02	5.0	3.02 ± 2.65	3.0	<0.001*
**GDS**	13.64 ± 3.59	13.0	12.42 ± 2.74	12.0	0.012*
**GHQ-28**	23.55 ± 10.3	22.0	20.66 ± 10.09	18.0	0.013*
**AIS**	6.76 ± 3.85	6.0	5.66 ± 4.19	4.0	0.012*
**STAI (X-1)**	41.64 ± 9.48	41.0	38.47 ± 10.08	38.5	0.022*
**STAI (X-2)**	41.85 ± 9.14	41.0	38.21 ± 8.93	37.5	0.007*
**CECS_anger**	17.15 ± 4.09	16.0	18.23 ± 3.5	18.0	0.036*
**CECS_depression**	17.93 ± 3.88	19.0	17.93 ± 3.84	18.0	NS
**CECS_anxiety**	16.57 ± 2.84	17.0	16.57 ± 2.83	17.0	NS
**CECS_total**	51.64 ± 9.24	52.0	52.73 ± 8.08	52.0	NS

AIS, Athens Insomnia Scale; CECS, Courtauld Emotional Control Scale; GDS, Geriatric Depression Scale; GHQ-28, General Health Questionnaire-28; MDQ, Mood Disorder Questionnaire; Me, median; NS, not significant; p, p-value; SD, standard deviation; SMAST-G, Short Michigan Alcoholism Screening Test—Geriatric Version; STAI, State-Trait Anxiety Inventory; $\bar{x}$, mean; *, statistically significant value statistically significant value (p < 0.05; Mann–Whitney U-test).

The results obtained with various scales were also stratified according to the economic status of the participants. Respondents with poor economic status presented with significantly higher levels of symptoms for non-psychotic mental disorders measured with GHQ-28 than the subjects with a good economic condition (p = 0.028). Furthermore, participants with poor economic status had significantly higher AIS, STAI X-1 and STAI X-2 scores compared with the respondents with either good (p = 0.044, p = 0.017 and p = 0.009, respectively) or very good financial condition (p = 0.015, p = 0.003 and p <0.001, respectively). Finally, persons with average economic status were shown to have higher levels of trait anxiety than those with a very good financial condition (p = 0.035) (**Table 6**).

**Table 6:** Effect of economic status on the scores of psychometric scales used in the study.

	Economic status								p
	Poor (I) N = 6		Moderate (II) N = 100		Good (III) N = 120		Very good (IV) N = 21		
	$\bar{x}$	Me	$\bar{x}$	Me	$\bar{x}$	Me	$\bar{x}$	Me	
**MDQ**	5.5 ± 2.59	5.0	3.88 ± 2.99	4.0	3.15 ± 2.75	3.0	3.38 ± 2.78	3.0	NS
**GDS**	14.67 ± 4.27	15.0	12.89 ± 3.16	12.0	12.55 ± 2.93	12.0	12.71 ± 2.61	12.0	NS
**GHQ-28**	30.67 ± 12.04	28.0	22.26 ± 9.94	20.5	20.91 ± 10.22	18.0	17.95 ± 9.57	15.0	I–IV: 0.028*
**AIS**	10.17 ± 3.54	11.0	6.36 ± 4.32	5.0	5.58 ± 3.74	5.0	5.05 ± 4.71	3.0	I–III: 0.044* I–IV: 0.015*
**STAI (X-1)**	51.67 ± 8.69	49.0	40.14 ± 9.55	40.0	38.8 ± 10.01	39.0	35 ± 9.63	34.0	I–III: 0.017* I–IV: 0.003*
**STAI (X-2)**	50 ± 4.6	50.0	40.52 ± 8.96	41.0	38.33 ± 9.04	37.0	34.71 ± 7.97	34.0	I–III: 0.009* I–IV: <0.001* II–IV: 0.035*
**CECS_anger**	20 ± 2.68	19.5	17.87 ± 4.07	17.0	17.93 ± 3.13	18.0	17.71 ± 4.93	16.0	NS
**CECS_depression**	19 ± 3.63	19.5	17.9 ± 3.69	18.0	18.03 ± 4.09	17.5	17.14 ± 3.18	17.0	NS
**CECS_anxiety**	17.83 ± 1.94	18.5	16.46 ± 3.02	17.0	16.56 ± 2.77	16.0	16.76 ± 2.43	16.0	NS
**CECS_total**	56.83 ± 5.88	58.0	52.23 ± 8.79	52.0	52.53 ± 8.14	52.0	51.62 ± 8.75	52.0	NS
**SMAST-G**	1.5 ± 2.74	0.5	1.39 ± 1.96	1.0	0.93 ± 1.5	0.0	1.33 ± 2.08	1.0	NS

AIS, Athens Insomnia Scale; CECS, Courtauld Emotional Control Scale; GDS, Geriatric Depression Scale; GHQ-28, General Health Questionnaire-28; MDQ, Mood Disorder Questionnaire; Me, median; NS, not significant; p, p-value; SD, standard deviation; SMAST-G, Short Michigan Alcoholism Screening Test—Geriatric Version; STAI, State-Trait Anxiety Inventory; $\bar{x}$, mean; *, statistically significant value (p < 0.05; Kruskal–Wallis ANOVA and appropriate post-hoc tests).

The scores were also analyzed according to the marital status of the respondents (**Table 7**). Group III, with 42 participants in total, included both divorcees and those who reported the separation, as the latter subgroup was very small. Singles had the highest AIS scores and the highest levels of anger control of all analyzed groups. The AIS score for the singles turned out to be significantly higher than for widows and widowers (p = 0.041), whereas the level of anger control among singles was significantly higher than in married respondents (p = 0.032). Divorcees presented with significantly lower levels of depression control according to the CECS than the married participants (p = 0.017). Moreover, married respondents had significantly lower levels of the overall index of emotion control than widowed (p = 0.022) and divorced participants (p = 0.023).

**Table 7:** Effect of marital status on the scores of psychometric scales used in the study.

	Marital status								p
	Married (I) N = 130		Widowed (II) N = 52		Divorced/in separation (III) N = 42		Single (IV) N = 23		
	$\bar{x}$	Me	$\bar{x}$	Me	$\bar{x}$	Me	$\bar{x}$	Me	
**MDQ**	3.31 ± 2.83	3.0	3.27 ± 2.88	4.0	4.17 ± 2.99	4.0	4.13 ± 2.77	4.0	NS
**GDS**	12.88 ± 2.92	12.0	12.63 ± 3.4	12.0	12.48 ± 3.18	11.0	12.83 ± 2.66	13.0	NS
**GHQ-28**	21.28 ± 8.66	20.0	20.52 ± 12.29	17.0	21.43 ± 12.21	17.5	24.48 ± 9.22	22.0	NS
**AIS**	5.95 ± 3.78	5.0	5.42 ± 4.43	3.0	5.52 ± 3.95	4.0	8.04 ± 5.09	7.0	II–IV: 0.041*
**STAI (X-1)**	40.05 ± 9.3	41.0	38.12 ± 11.29	37.0	38 ± 11.39	37.5	40.48 ± 7.87	40.0	NS
**STAI (X-2)**	39.35 ± 8.7	39.0	38.81 ± 10.35	37.5	37.6 ± 9.71	35.5	42.09 ± 6.85	42.0	NS
**CECS_anger**	17.26 ± 3.32	17.0	18.62 ± 4.32	19.0	18.26 ± 3.42	18.5	19.65 ± 3.97	19.0	I–IV: 0.032*
**CECS_depression**	17.17 ± 3.68	16.0	18.62 ± 4.45	19.0	19.12 ± 3.56	19.0	18.48 ± 2.94	19.0	I–III: 0.017*
**CECS_anxiety**	16.16 ± 2.78	16.0	17.12 ± 3.18	17.5	17.14 ± 2.48	17.0	16.57 ± 2.66	17.0	NS
**CECS_total**	50.59 ± 7.76	50.5	54.35 ± 9.79	55.0	54.52 ± 7.35	55.0	54.7 ± 8.51	55.0	I–II: 0.022* I–III: 0.023*
**SMAST-G**	1.15 ± 1.63	1.0	0.79 ± 1.68	0.0	1.43 ± 2.07	1.0	1.57 ± 2.23	0.0	NS

AIS, Athens Insomnia Scale; CECS, Courtauld Emotional Control Scale; GDS, Geriatric Depression Scale; GHQ-28, General Health Questionnaire-28; MDQ, Mood Disorder Questionnaire; Me, median; NS, not significant; p, p-value; SD, standard deviation; SMAST-G, Short Michigan Alcoholism Screening Test—Geriatric Version; STAI, State-Trait Anxiety Inventory; $\bar{x}$, mean; *, statistically significant value (p < 0.05; Kruskal–Wallis ANOVA and appropriate post-hoc tests).

Men significantly more often (44%) than women (25%) had SMAST-G scores raising suspicion of alcohol-related problems (**Table 8**).

**Table 8:** Effect of gender on the occurrence of possible problem alcohol drinking among the study respondents.

	Men		Women		p
	n	%	n	%	
**SMAST-G** **(2 pts and more)**	14	44%	53	25%	0.023*
**SMAST-G** **(<2 pts)**	18	56%	162	75%	
**Total**	32	100%	215	100%	

p, p-value; SMAST-G, Short Michigan Alcoholism Screening Test—Geriatric Version; *, statistically significant value (p < 0.05; Mann–Whitney U-test).

The results obtained with various scales were also compared between the groups identified based on other sociodemographic variables, i.e. gender, age, education and place of residence. The results are not shown in this paper, as none of these comparisons demonstrated statistically significant differences.

During the next stage of the analysis, correlations between the scores of various scales were analyzed. The results for the entire study group are shown in **Table 9**. The scores of all scales included in the table correlated positively with one another. Particularly important seem to be the correlations of STAI-X1 and STAI-X2 scores with the GHQ-28 and AIS scores. Moreover, a strong significant positive correlation was found between the AIS and GHQ-28 scores. The table does not contain the CECS scores and the values of its subscales as no significant correlations were found between them and the other scales.

**Table 9:** Spearman’s coefficients of rank correlation between the scores of validated psychometric scales used in the study.

		SMAST-G	STAI (X-2)	STAI (X-1)	AIS	GHQ-28	GDS
**MDQ**	**r**	0.252	0.295	0.215	0.218	0.195	0.258
	**p**	<0.001*	<0.001*	<0.001*	<0.001*	0.002*	<0.001*
**GDS**	**r**	0.215	0.500	0.462	0.318	0.404	–
	**p**	<0.001*	<0.001*	<0.001*	<0.001*	<0.001*	
**GHQ-28**	**r**	0.147	0.710	0.714	0.676	–	–
	**p**	0.021*	<0.001*	<0.001*	<0.001*		
**AIS**	**r**	0.159	0.625	0.577	–	–	–
	**p**	0.013*	<0.001*	<0.001*			
**STAI (X-1)**	**r**	0.16	0.809	–	–	–	–
	**p**	0.009*	<0.001*				
**STAI (X-2)**	**r**	0.212	–	–	–	–	–
	**p**	0.001*					

AIS, Athens Insomnia Scale; GDS, Geriatric Depression Scale; GHQ-28, General Health Questionnaire-28; MDQ, Mood Disorder Questionnaire; p, p-value; r, Spearman’s rank correlation coefficient; SMAST-G, Short Michigan Alcoholism Screening Test—Geriatric Version; STAI, State-Trait Anxiety Inventory; *, statistically significant value (p <0.05; Kruskal–Wallis ANOVA and appropriate post-hoc tests).

**Figure 1:**
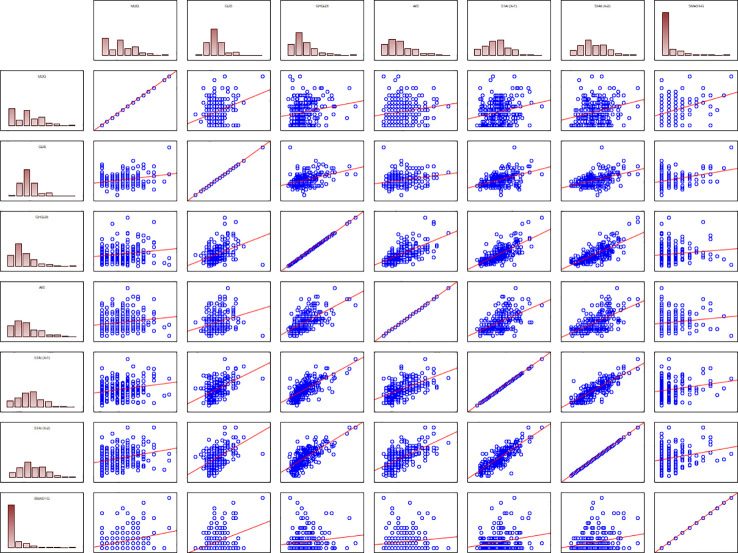
Distribution plots illustrating correlations between psychometric scales used in the study.

Graphic illustration of the results mentioned above, with the distributions of various scales’ scores against the results obtained with other scales, is shown in **Figure 1**.

The analysis of correlation was also carried out after stratification of the results according to the demographic variables. The results of the subgroup analysis were generally consistent with those for the entire study group. A significant inverse correlation (r = −0.416, p = 0.018) between the levels of anger control and MDQ scores was found among men. Among women, the correlation between these two variables was weaker, albeit still significant (r = −0.154, p = 0.024). A significant relationship between the level of anger control and MDQ scores was also observed in singles (r = −0.613, p = 0.002), but not in other marital status groups. Furthermore, the singles showed significant inverse correlations between MDQ score, depression control (r = −0.462, p = 0.027) and the overall index of emotion control (r = −0.536, p = 0.008). Similar relationships were not found in other marital status groups.

## Discussion

A few published studies dealt with the psychological wellbeing of the elderly without previously diagnosed mental disorders. However, nothing is known about the mental health of active seniors participating in the Third Age University program. This means that there is a considerable knowledge gap in this matter, both in Poland and globally. Meanwhile, the data about the psychological wellbeing of the elderly seem to be vitally important in the context of some specific preventive activities.

### Presence of Possible Bipolar Disorder (MDQ)

Mean MDQ score documented in this study (3.52 ± 2.87 pts) implied that the respondents were unlikely to present with bipolar disorders. Indeed, the proportion of the study group who screened positively for possible bipolar disorder was only 4.05%. Mean MDQ score in the study conducted by Altınbaş et al. (35) was 4.1 ± 3.0 pts, with 29.0% of the respondents screening positively for possible bipolar disorder. In another study, conducted by Kim et al. (36), the percentage of possible bipolar disorder was estimated at 4.3%. Other authors also reported similar frequencies of possible bipolar disorder; for example, Hirschfeld et al. (37) (3.7%). Mean MDQ score in an Italian study of 143 geriatric patients was 7.24 ± 1.75 pts (38), and according to Vöhringer et al. (39), mean MDQ score for 197 patients from 10 primary healthcare centers amounted to 4.74 ± 2.70 pts. A reason behind the lower MDQ score in our respondents might be a smaller size of the sample. However, our results seem to be consistent with those published by other Polish authors. In the study conducted by Pawłowski et al. (40), mean MDQ score for the whole study group was 3.57 ± 3.36 pts. In another study, Rybakowski et al. (41) reported a mean MDQ score of 3.6 ± 3.2 pts, and the scores for 20% of the respondents exceeded the cut-off value for possible bipolar disorder.

Castilla-Puentes et al. (42) used the MDQ to examine 1,505 respondents and found no statistically significant differences in the age, gender, economic status and education of patients with possible bipolar disorder and without. These findings are consistent with the results of our present study.

### Occurrence of Depression (GDS)

Mean GDS score for our study participants was 12.75 ± 3.03 pts, which was a markedly higher value than those documented previously in the elderly. In an Italian study of 143 geriatric patients, mean GDS score was 3.51 ± 3.38 pts (38), and the score in a Nigerian study of 458 older persons equaled 4.15 ± 4.80 pts (43). Interestingly, the level of depression in our group of active seniors was higher than in either the general population of the elderly or specific groups of geriatric patients. One potential explanation might be a broader age range of our participants than those examined by other authors, and a large proportion of respondents aged 60 to 69 years, i.e. at the age when many life breakthroughs occur (e.g. withdrawal from occupational activities and retirement). Moreover, it should be stressed that our study group was substantially smaller than those included in previous studies, and unlike other authors, we used the full 30-item version of the GDS. Another potential reason behind the higher levels of depression in our respondents might be the fact that they completed the survey online, without a detailed instruction on how to fill in the questionnaires, other than a brief introduction attached to each scale.

More than 90% of the respondents participating in this study presented with mild depression, and in more than 3%, the GDS score corresponded to moderate or severe depression. According to Tiwari et al. (44), depression was present in most of 45 elderly residents of nursing homes participating in their study (50.0% of men and 28.0% of women). In contrast, in another study conducted with a validated self-report scale by Sjöberg et al. (45), the frequency of depression was estimated at 9.1%.

In our present study, the GDS scores increased with the age of the respondents. While some authors observed an opposite relationship between the depression and age (46) or found no association at all (47), our findings seem to be consistent with the results of most previous studies (6, 45, 48). A meta-analysis conducted by Zhao et al. (49) showed that the prevalence of depression increased with age among persons aged 55 to 90 years, but not in those older than 90 years.

Our observation about the markedly higher prevalence of depression among women than in men is consistent with the results of other studies conducted worldwide (45, 46, 49). The less frequent occurrence of depression among men may be partially associated with gender stereotypes (for example, men less often admit having depressive symptoms because depression is perceived by some as a feminine problem, and thus, not matches a masculine stereotype) (50). Moreover, Poland is characterized by a substantial overrepresentation of older women over older men, which also might contribute to the relationship observed in our study. Another potential explanation might be the fact that in a traditional model, women are primarily responsible for house chores, and hence, to a certain degree economically dependent on men.

We did not find a statistically significant association between marital status and the level of depression measured with the GDS. The lack of such a relationship was also reported previously by Sjöberg et al. (45).

Carta et al. (38) found an inverse correlation between GDS and MDQ scores. In the study conducted by Amin-Esmaeili et al. (51), 224 patients (3% of the study sample) both presented with severe depression and satisfied the criteria of a possible bipolar disorder according to the MDQ. The frequency of diagnosing both problems in a single patient was similar regardless of gender. A study of patients with personality disorders demonstrated that this group presented with recurrent depressive disorders and abnormal MDQ scores more often than the healthy controls. Normal MDQ scores were found in 63% of the respondents with personality disorders and 82.5% without (52).

Our study also demonstrated a significant association between the severity of depression and insomnia, which is consistent with the results published by Chinese authors (53).

### General Psychological Wellbeing (GHQ-28)

Mean GHQ-28 score of our respondents was 21.44 ± 10.21 pts, implying that the participants of the Third Age University program were free from non-psychotic mental disorders. The GHQ-28 score in our group was similar to those obtained in previous studies conducted in Poland and abroad. Andruszkiewicz et al. (54) examined 219 respondents, among them 106 in late adulthood, 88 in early and 25 in late old age; the highest GHQ-28 scores (27.37 ± 17.43 pts) were found in the youngest group, and the lowest (22.60 ± 11.45 pts) in younger old. Hence, that study showed that these were persons in late adulthood, rather than the elderly, who presented with the worst psychological wellbeing. In another Polish study, conducted by Pytel et al. (55) in a group of patients with rheumatoid arthritis (mean age 57 years), mean GHQ-30 score was 19.23 ± 13.33 pts.

Despite a relatively high mean GHQ-28 score, approximately one-third of our study group satisfied the criteria for possible non-psychotic mental disorders. The proportion of patients who satisfied those criteria was higher than reported previously in China (23.8%) (56) and Finland (15.3%) (57), but lower than in Brazil (38.5%) (58) and Japan (36.9%) (59). The discrepancies between the studies might result from differences in sampling methodology, definitions of poor mental health, research instruments and follow-up time.

Unlike in our present study, Nagasu et al. (59) found significant gender-related differences in GHQ-12 scores. In turn, Wang et al. (56) reported a relationship between marital status and mental health, which was not observed in our present study. Additionally, the latter authors found correlations between the psychological wellbeing measured with GHQ-12, place of residence and economic status. We did not observe a significant association between the place of residence and manifestations of non-psychotic mental disorders. However, similar to Wang et al. (56), we found a relationship between the presence of such symptoms and economic status.

### Occurrence of Insomnia (AIS)

Mean AIS score in this study was 5.96 ± 4.12 pts. The symptoms of insomnia were present in nearly 45% of the Third Age University program participants. In our previous study of the elderly (60), mean AIS score was higher, 7.20 ± 6.00 pts, and thus, corresponded to the occurrence of sleep disorders. Nearly 55% of the respondents scored 6 pts or higher, and hence, were likely to suffer from insomnia. Uchmanowicz et al. (61) found the symptoms of insomnia in 59% of geriatric patients with arterial hypertension. In the study conducted by Kim et al. (62) among 881 Koreans aged 60 years and older, the frequency of insomnia measured with the AIS was estimated at 32.7%. Mean AIS score for 62 older persons examined by Ibáñez-del Valle et al. (63) was 4.0 ± 4.0 pts. According to Abd Allah et al. (64), the prevalence of insomnia measured with the AIS in the group of 107 seniors from Zagazig (Egypt) was 33.6%. Insomnia was found in up to 47.9% of Polish patients with arterial hypertension examined by Prejbisz et al. (65). The prevalence of insomnia in a group of 142 persons examined by Hishikawa et al. (66) was 17.1%. In a Greek study, conducted by Paparrigopoulos et al. (67) in a group of 1,005 participants, the frequency of insomnia measured with the AIS was estimated at 25.3%. Potential reasons behind the discrepancies in the prevalence of insomnia documented by various authors might include differences in the quality of healthcare, low awareness of the availability of specialists in sleep disorders management among the elderly, and presence of chronic somatic comorbidities. Alternative explanations for the between-study discrepancies include cultural differences, as well as confounding effects of environmental and lifestyle factors.

In our present study, singles presented with significantly higher AIS scores than widows and widowers. Also, previous studies conducted in Egypt (64) and China (68) demonstrated that persons living alone suffered from insomnia more often than other respondents.

### The Level of Emotion Control (CECS)

Mean overall index of emotion control for the study group was 52.43 ± 8.41 pts. In our other study (69), mean overall CECS score was slightly higher and amounted to 53.74 ± 8.55 pts, which is considered a moderate ability to suppress negative emotions. In the study conducted by Symonides et al. (70), mean CECS score for hypertensive patients was 54.0 ± 12.0 pts. Hence, the mean CECS score for participants of our present study was similar to the results mentioned above and consistent with the value for the general Polish population (50.0 ± 11.00 pts) (30). This implies that our sample provided adequate representation for the Polish seniors. According to Głębocka et al. (71), mean levels of emotion control in patients with obstructive sleep apnea and healthy controls were 16.5 ± 4.8 and 16.9 ± 3.7 pts, respectively. Markedly lower levels of emotion control documented in that study might be associated with a smaller sample size (N = 57), as well as with the fact that the CECS scores were determined in patients with a specific disease entity, i.e. obstructive sleep apnea (71).

In our previous study (69), a positive correlation was found between the level of anger control (CECS) and trait anxiety (STAI X-2) (r = 0.307, p = 0.002) in the subgroup of participants from the Healthy Senior University program. This means that the better the subjective ability to suppress anger among the respondents from the program, the higher the level of trait anxiety in this group. However, in our present study, no correlation was observed between the levels of anger control and trait anxiety, as well as between any of the CECS scores and other analyzed scales.

### Anxiety Levels (STAI)

Mean state anxiety (STAI X-1) and trait anxiety (STAI X-2) scores for the study group were 39.99 ± 10.00 and 39.19 ± 9.11 pts, respectively, which corresponds to moderate anxiety levels. Mean levels of state and trait anxiety in the study conducted by Aggelopoulou et al. (72) were 54.5 ± 9.4 and 52.8 ± 8.5 pts, respectively. In our previous study (69), mean state (STAI X-1) and trait anxiety (STAI X-2) scores were 48 and 49 pts, respectively, which is 10 pts higher on average than in the present study. The difference might be associated with the fact that a certain proportion of participants in our previous study were the residents of social welfare homes whose anxiety levels might be higher. In the study of Lancon et al. (73), mean STAI score was 43.7 pts. According to Hosseini et al. (74, 75) the STAI scores ≥40 pts were found in 145 (50.9%) patients examined by this group. In the study conducted by Remröd et al. (76), mean levels of state and trait anxiety were 38.0 ± 12.2 and 36.5 ± 11.9 pts, respectively. Unlike the other authors mentioned above, Di Mattei et al. (77) reported slightly higher levels of state anxiety (42.7 ± 10.1 pts) than trait anxiety (39.8 ± 8.06 pts). Compared with the results mentioned above, the levels of anxiety documented in our present study were relatively high. This might be associated with the exposure of our respondents to chronic stress, e.g. because of their family situation, loneliness, death of a spouse or other relatives, etc.

### Problem Alcohol Drinking (SMAST-G)

Unlike for younger persons, little is known about alcohol abuse, its harmful effects and relevant preventive measures among the elderly. Generally, the alcohol-related harm in this age group is considered to be lower, as older persons typically drink less than the younger ones, are less prone to high-risk drinking and harmful health effects of alcohol abuse. However, the patterns and determinants of alcohol consumption seem to be similar as in the younger population. Nevertheless, we know little about alcohol abuse among the elderly, as the representation of this age group in nation-wide studies dealing with the problem in question is usually too low. This justifies further research in this matter (78).

Up to one-fourth of the elderly participating in our present study were likely to experience problem alcohol drinking based on their SMAST-G scores. In one previous study, the prevalence of alcohol abuse among older Indigenous Americans was estimated at 46% among men and 18% among women (79). These figures were markedly lower than the occurrence of alcohol-related problems among American citizens aged 45 years or older (63.0 and 40.08% for men and women, respectively). The prevalence of alcohol abuse in men and women participating in Strong Heart Study carried out in North and South Dakota was 60.0 and 37.7%, respectively (80). The discrepancies in the frequency of problem alcohol drinking documented in various studies might be among others associated with the evaluated timeframe of alcohol consumption (last month vs. last year).

### Limitations of the Study

Our present study has some potential limitations. First, this was a cross-sectional study based solely on a self-report survey. Although the scales used in this study are sensitive instruments designed to detect various psychological disorders, they all center on subjective manifestations, rather than objective clinical criteria, which poses a risk of false-positive results. Second, the study group was too small to generalize the results onto the entire population of active seniors participating in the Third Age University program. Third, there was an overrepresentation of women in the study group, and hence, the results should be verified in an equally large group of men. However, both the actual percentage of people studying at the Third Age Universities as well as the actual demographic trends in the Polish population is distinguished by the high proportion of women relative to men. Additionally, the occurrence of chronic diseases determines the mental state of older people, but this aspect was not the subject of our study. We focused on the overall assessment of the mental health status of older people attending of the Third Age Universities. As a research team, we will include them in our future research protocols. Despite these limitations, the results of this study might constitute a starting point for further research on the mental health of the Third Age University program participants and its sociodemographic determinants. Optimally, these questions should be addressed in a longitudinal study.

In conclusion, the study group was characterized primarily by the mild depressive and anxiety symptoms. The mental health of the Polish participants of the Third Age University program was significantly modulated by their socio-occupational and marital status, and financial condition. The study showed that persons with likely problem alcohol drinking differed from other respondents in terms of the occurrence of possible bipolar disorder, depression, and non-psychotic symptoms of mental disorders, state and trait anxiety levels and anger control. There is a need for further research in the field of mental health status in the Third Age Universities seniors to determine the prevalence of these phenomena on a national scale.

## Data Availability Statement

The raw data supporting the conclusions of this article will be made available by the authors, without undue reservation.

## Ethics Statement

The studies involving human participants were reviewed and approved by the Bioethics Committee of the Medical University in Bialystok (statute no. R-I-002/592/2019). The patients/participants provided their written informed consent to participate in this study.

## Author Contributions

Conceptualization: MC, EK-K, UC and KK. Data curation: MC and ŁC. Formal analysis: MC, ŁC, EK-K, UC, and KK. Funding acquisition: MC. Investigation: MC, ŁC, and KK. Methodology: MC, EK-K, and UC. Project administration: MC. Writing—original draft: MC and UC. Writing—review and editing: EK-K. All authors contributed to the article and approved the submitted version.

## Conflict of Interest

The authors declare that the research was conducted in the absence of any commercial or financial relationships that could be construed as a potential conflict of interest.
